# Rheumatoid arthritis-associated aortitis: a case report and literature review

**DOI:** 10.1186/2193-1801-3-509

**Published:** 2014-09-09

**Authors:** Shunta Kaneko, Hiroyuki Yamashita, Yusuke Sugimori, Yuko Takahashi, Hiroshi Kaneko, Toshikazu Kano, Akio Mimori

**Affiliations:** Division of Rheumatic Diseases, National Center for Global Health and Medicine, 1-21-1 Toyama, Shinjuku, Tokyo, 162-8655 Japan

**Keywords:** Rheumatoid arthritis, Rheumatoid vasculitis, Aortitis

## Abstract

Rheumatoid arthritis (RA) is a systemic autoimmune inflammatory disorder that primarily affects the synovial joints. Rheumatoid vasculitis (RV) is an extra-articular manifestation of RA, and its association with aortitis is rare and not widely recognised. Here, we report the case of a 69-year-old woman with RA-associated aortitis and review the literature on rheumatoid aortitis. The mean oral steroid dose administered to RA-associated aortitis patients was 46.3 mg/day prednisolone (PSL). In our patient, the aortitis was also thought to be due to RV because she had findings of RV, such as cutaneous ulceration and a high rheumatoid factor titre, and because a moderate PSL dose dramatically improved the clinical findings. RA-associated aortitis, if left untreated, can be fatal; therefore, early detection and treatment initiation is very important.

## Introduction

Aortitis is a vasculitis that affects large arteries more often than other vasculitides. According to the Chapel Hill Consensus Conference (CHCC) 2012 definitions, Takayasu arteritis (TKA) and giant cell arteritis (GCA) are the two major variants (Jennette et al. 2013). Other autoimmune diseases associated with aortitis are ankylosing spondylitis (AS), Behçet’s disease, rheumatoid arthritis (RA), and systemic lupus erythematosus (SLE). In addition, aortitis is associated with infectious diseases, such as syphilis and tuberculosis.

Rheumatoid arthritis is an autoimmune and systemic inflammatory disorder mainly affecting the synovial joints. Sometimes, RA patients have extra-articular manifestations, such as rheumatoid vasculitis (RV). The concept of RV started to evolve in the 1960s when vasculitis with significant clinical manifestations was described in RA patients (Schmid et al. 1961; Bywaters and Scott 1963; Wikinson and Torrance 1967). The clinical manifestations of vasculitis are varied and include weakness, weight loss, skin rashes, cutaneous ulcerations, gangrene, peripheral neuropathy, and visceral infarction; approximately 90% of RA patients with RV have skin manifestations (Jennette et al. 2013). The association of aortitis with RV is rare and is not widely recognised. RA aortitis was originally described by Mallory in 1936 (Mallory 1936) and has been noted in earlier reports (Schmid et al. 1961; Bywaters and Scott 1963; Wikinson and Torrance 1967). In 45 cases of active non-infectious aortitis seen among 513 consecutive ascending aortic resections, approximately 4% were RA patients, as reported by Miller et al. ( 2006).

Here, we describe a case of aortitis associated with RA and review the literature.

## Patient and methods

We report a case of aortitis associated with RA and reviewed 24 cases among 13 publications on RA aortitis. To identify published cases of RA aortitis, we performed a PubMed search using the following terms: “rheumatoid arthritis”, “autoimmune disorder”, “aortitis”, “Takayasu aortitis”, and “large vessel vasculitis”.

We evaluated epidemiological data such as the age at onset of RA and aortitis, interval between the onset of RA and that of aortitis, pathology and laboratory findings, and human leukocyte antigen (HLA) type. The ethics committee of our institute approved this study.

## Case report

In April 2010, a 69-year-old woman presented to our hospital with pain and swelling in the small joints of her hands for the past 2 months. The laboratory examination revealed an elevated erythrocyte sedimentation rate (ESR; 31 mm/h) and C-reactive protein (CRP; 1.69 mg/L) level. Her rheumatoid factor (RF) and anticyclic citrullinated peptide (anti-CCP) levels were 215.4 and 51.1 IU/mL, respectively. Subsequent radiography showed interstitial pneumonia (nonspecific interstitial pneumonia [NSIP] pattern), which was suggestive of RA. From these findings, the patient was diagnosed with RA according to the 2010 American College of Rheumatology (ACR)/European League Against Rheumatism (EULAR) RA Classification Criteria (8 points total: joint involvement 3 points, serology 3 points, acute-phase reactants 1 point, duration of symptoms 1 point). RA treatment was initiated with 1,000 mg/day salazosulfapyridine (SASP), which improved her clinical symptoms and laboratory findings. Based on the clinical course, however, SASP was not sufficiently effective after 1 year of treatment; consequently, the treatment was supplemented with 3 mg/day tacrolimus. Subsequently, her articular symptoms and laboratory inflammatory findings resolved.

**Figure 1:**
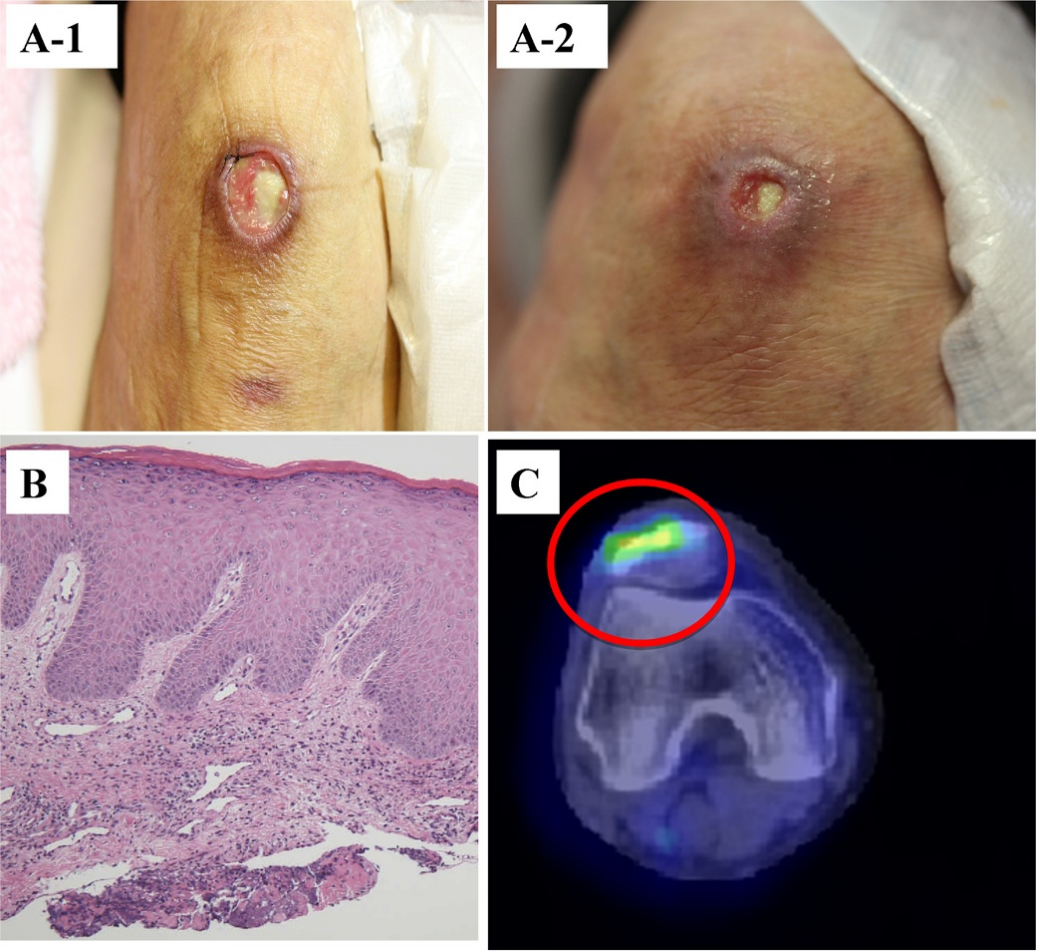
**Cutaneous ulceration before and after steroid therapy; pathology and PET/CT findings of the cutaneous ulceration. (A-1)** The right knee cutaneous ulcer measured 4 cm before steroid therapy. **(A-2)** One month after the treatment, it measured < 1 cm and showed granulation tissue growth. **(B)** A biopsy of the edge of the ulcer revealed a mild lymphocytic infiltrate in the superficial dermis; however, no obvious findings of vasculitis were observed. **(C)** FDG PET/CT demonstrated increased tracer uptake in the ulcer, suggesting inflammation.

**Figure 2:**
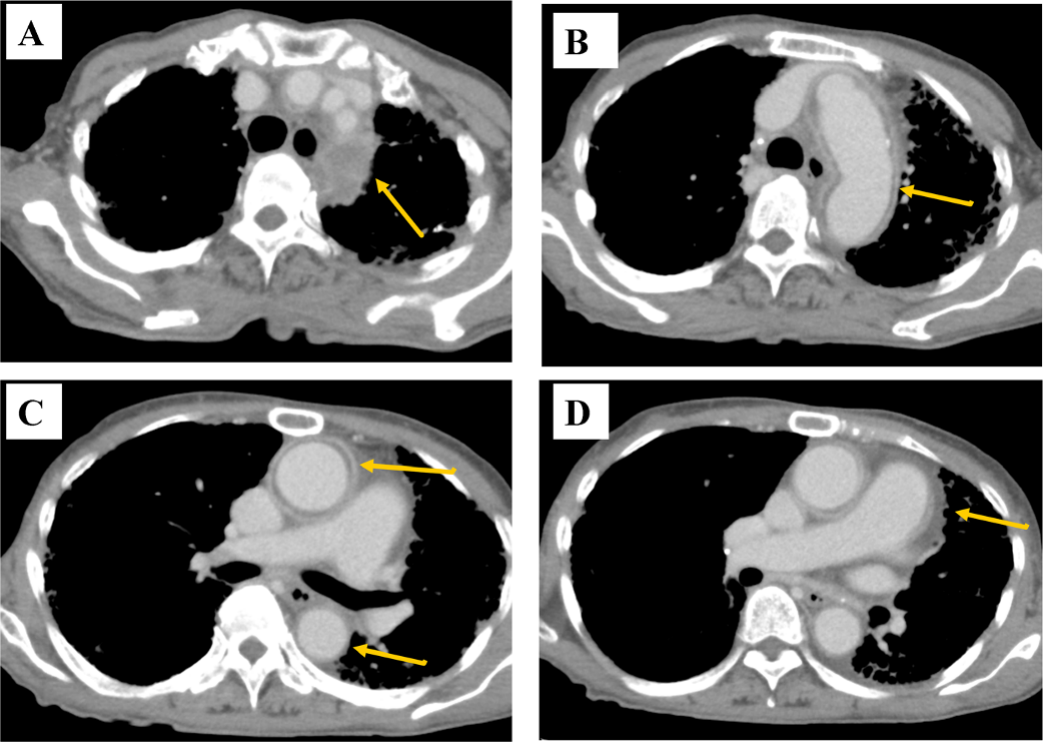
**Thoracic contrast enhanced CT findings of aortitis before steroid therapy.** Thoracic contrast-enhanced CT demonstrated thickening of the aortic wall at each following level with a contrast effect, and the non-contrast layered area on the lumen. **A**. brachiocephalic artery and left subclavian artery. **B**. aortic arch. **C** and **D**. ascending aorta.

**Figure 3:**
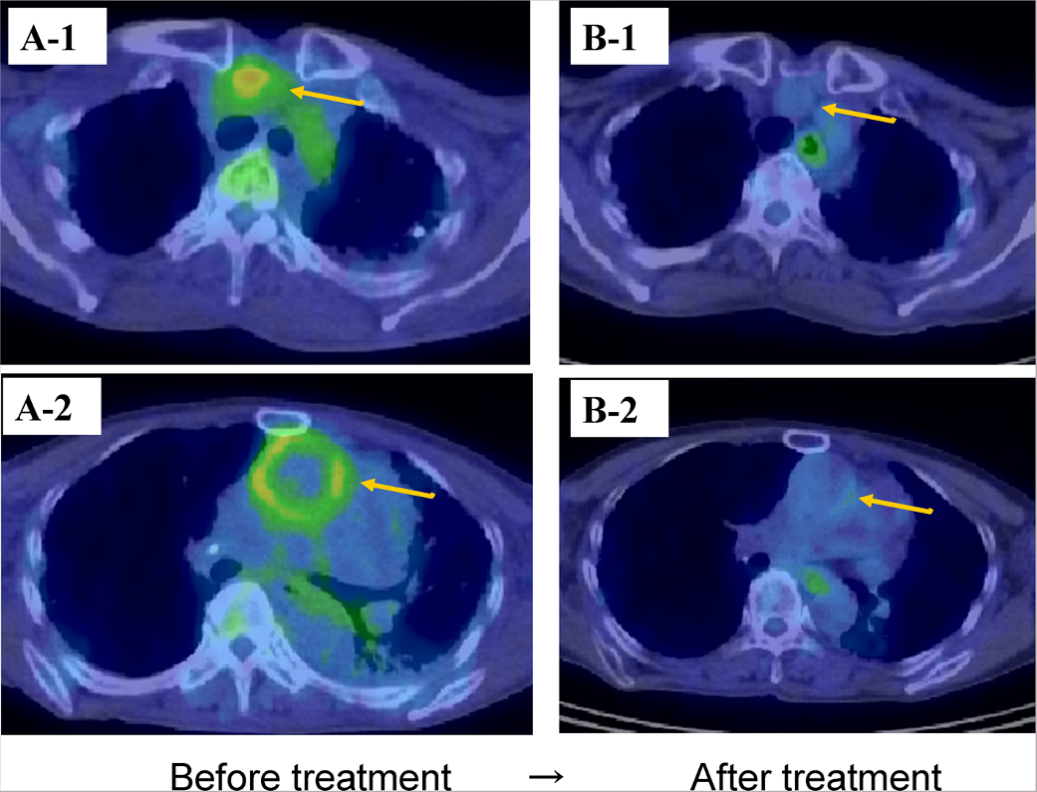
**FDG-PET/CT findings of aortitis before and after steroid therapy. A-1** and **A-2**. FDG PET/CT showed increased tracer uptake in the ascending aorta, aortic arch, brachiocephalic artery, and left subclavian artery. **B-1** and **B-2**. After steroid therapy, FDG-PET/CT showed a significant reduction in the FDG uptake in the same area.

In January 2014, she visited our outpatient clinic with a fever of 38–39°C, cough, and upper back pain for the past 1 week. She also had a skin ulcer over the right patella that had appeared 1 month earlier (Figure 1A-1). The laboratory evaluation revealed elevated inflammatory markers (ESR 119 mm/h and CRP 14.99 mg/dL). Her rheumatoid factor (RF) and matrix metalloproteinase-3 (MMP-3) levels were 2,010.0 IU/mL and 122.4 IU/L, respectively. The C3 and C4 levels were reduced to 122.0 and 12.7 mg/dL, respectively. Cytoplasmic pattern antineutrophil cytoplasmic antibodies (C-ANCA), perinuclear ANCA (P-ANCA), antinuclear antibodies, anti-DNA, anti-Sm, RNP, Scl-70, and Ro and La antibodies were all negative. Three sets of blood cultures were negative. The rapid plasma reagin (RPR) test, *Treponema pallidum* haemagglutination assay (TPHA), QuantiFERON test (QFT), galactomannan antigen, and β-D-glucan were negative. An echocardiogram showed no evidence of vegetations in her heart. Thoracic-abdominal computed tomography (CT) was performed to investigate the persistently elevated inflammatory markers and showed no evidence of pneumonia, while dilation of the ascending and descending aorta, aortic arch, brachiocephalic artery, and left subclavian artery were noted (Figure 2). In addition, there was no evidence of atherosclerosis or aneurysm or the risk factors for bacterial aneurysm. Subsequently, fluorodeoxyglucose-positron emission tomography/CT (FDG-PET/CT) showed strong abnormal tracer uptake consistent with the dilated aortas on CT (Figure 3A). Moreover, FDG uptake was observed in the skin ulcer on the knee (Figure 1B). Although skin from the ulcer was biopsied, no obvious vasculitis findings were observed (Figure 1C).

**Figure 4:**
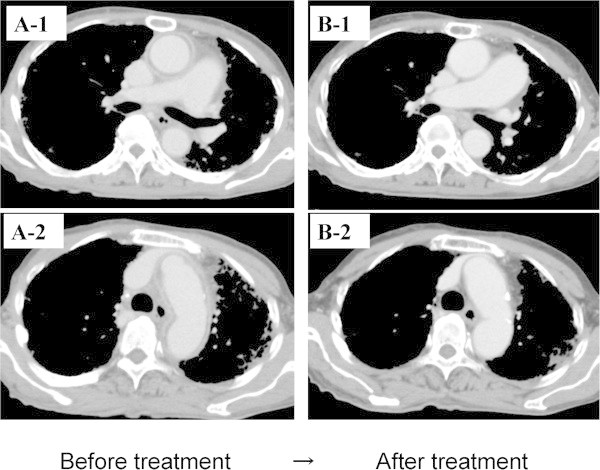
**Thoracic contrast-enhanced CT findings of aortitis before and after steroid therapy. A-1 and A-2.** Before steroid therapy, the thoracic contrast-enhanced CT demonstrated thickening of the aortic wall at the level of the ascending aorta and aortic arch, brachiocephalic artery, and left subclavian artery with a contrast effect. **B-1** and **B-2**. After steroid therapy, the thickening of the aortic wall had disappeared completely.

First, anti-bacterial therapy was initiated; however, this did not resolve the fever or the elevated ESR and CRP and did not decrease the dilation of the aorta on radiography. Therefore, we suspected autoimmune aortitis and initiated steroid therapy (prednisolone [PSL], 30 mg/day) 7 days after initiating the antibiotic therapy. One day after steroid therapy was started, the patient’s fever abated, her upper back pain disappeared, and the inflammation markers began to decrease. The right knee skin ulcer also shrank markedly (Figure 1A-2). These abnormalities returned to normal within 1 month after initiating steroid therapy. On day 28 following steroid therapy initiation, follow-up CT showed that the dilation of the aorta had disappeared completely (Figure 4). In addition, on day 42 after steroid initiation, FDG-PET revealed that the abnormal intense tracer uptake by the aorta had also disappeared (Figure 3B1-2). After the prednisolone dose was reduced to 27.5 mg/day, the patient was discharged.

## Literature review and assessment of our case

We have reported a case of aortitis associated with RA and reviewed 24 cases from 13 studies of RA aortitis (Table 1) (Sandring and Weil 1961; Falicov and Cooney 1964; Reimer et al. 1976; Rush et al. 1986; Sketchler and Waxman 1987; Mimura1 and Sueishi 1989; Gravallese et al. 1989; Towned et al. 1991; Nakabayashi et al. 1998; Korkmaz et al. 2001; Miller et al. 2006; Verweij1 et al. 2012; Mariani and Alexander 2013). As shown in Table 1, the mean patient ages at the times of diagnosis of RA and aortitis were 49 ± 14.5 (range 16–74) and 56 ± 15.2 (range 16–82) years, respectively. The average time elapsed between the onset of RA and the development of aortitis was 6.4 ± 8.68 (range −11 to 26) years. Extra-aortic arteritis involvement was found in 11/21 cases (52.4%). Regarding the pathology findings of RA aortitis, granulomas were found in 6/14 cases (42.9%), giant cells in 3/14 cases, and atherosclerosis in 14/18 cases (77.8%). Regarding the laboratory findings, RF was positive in 20/23 cases (87.0%), and rheumatoid nodules were noted in 11/21 cases (52.4%). The mean oral steroid dose for aortitis in the seven patients for whom the dose was reported was 46.3 (range 0–100) mg/day PSL. In addition, two patients were given intravenous steroids, one patient was given a single course of steroid pulse therapy with methyl-PSL 250 mg followed by a post-treatment 40 mg PSL dose, and one patient was given intravenous PSL 100 mg and intravenous cyclophosphamide (IVCY). The RA treatments prior to onset of aortitis varied.

**Table 1:** **Clinical characteristics of 25 patients with RA aortitis**

Case	Ref	Age/Sex	Disease duration of RA	Extra-aortic involvement of arteritis	Pathology of Aortitis			Rheumatoid nodule	RF	Type of HLA	RA treatment	Aortitis treatment
					Granuloma	Giant cells	Atherosclerosis					
1	6	68/F	ND	ND	–	+	–	ND	ND	ND	NSAIDs	NSAIDs
2	6	71/F	ND	ND	–	+	+	ND	ND	ND	NSAIDs	NSAIDs
3	7	63/F	23	–	ND	ND	+	ND	+	ND	ND	Steroid
4	7	65/F	10	ND	ND	ND	+	+	+	ND	ND	Steroid
5	8	16/F	0	+	ND	ND	ND	–	–	ND	Steroid	NSAIDs
6	9	49/F	5	+	+	+	–	+	+	ND	NSAIDs, Gold, PSL	Digitalis
7	10	37/F	−11^*^	–	ND	ND	ND	+	+	DR4	NSAIDs, CQ, Gold	PSL 40 mg
8	11	53/F	0	+	ND	ND	ND	–	–	ND	NSAIDs, HCQ	PSL 60 mg
9	12	50/F	0	–	–	–	–	–	+	ND	NSAIDs	–
10	13	46/M	9	+	–	–	+	+	+	ND	Aspirin	–
11	13	52/F	26	+	–	–	+	+	+	ND	Steroid, Aspirin	–
12	13	60/M	12	+	–	–	+	+	+	ND	Steroid, HCQ, Gold, CYC	–
13	13	61/F	11	–	+	–	+	+	+	ND	Steroid, Gold, Aspirin	High-dose steroid
14	13	61/F	3	–	+	–	+	+	+	ND	Steroid, HCQ, Gold	–
15	13	64/F	3	–	+	–	+	+	+	ND	Steroid, HCQ, Gold	–
16	13	67/M	2	+	–	–	+	+	+	ND	Steroid	–
17	13	68/M	21	+	+	–	+	+	+	ND	Steroid, HCQ	–
18	13	69/M	1	+	+	–	+	+	+	ND	Steroid, Gold, SASP	–
19	13	82/F	8	+	–	–	–	–	–	ND	Steroid, HCQ	Low-dose steroid
20	14	44/M	ND	+	ND	ND	ND	+	+	ND	Steroid, SASP	PSL 100 mg, IVCY 5 mg/kg
21	15	64/F	4	–	ND	ND	+	+	+	DR2,12(5)	ND	PSL 10 mg
22	16	36/F	2	–	ND	ND	ND	ND	+	DR4,1	SASP, MTX	PSL 50 mg
23	17	49/F	1	–	ND	ND	ND	–	+	ND	Steroid, MTX, LEF	PSL 40 mg, bosentan
24	18	43/F	ND	–	ND	ND	ND	–	+	ND	MTX, ADA	mPSL 250 mg, PSL 40 mg
25	Present case	73/F	4	+	ND	ND	+	–	+	B51, B52	SASP, TAC	PSL 30 mg

*Abbreviations*: *M* male, *F* female, *+* positive, *−* negative, *RA* rheumatoid arthritis, *RF* rheumatoid factor, *HLA* human leukocyte antigen, *ND* not described, *NSAIDs* non-steroidal anti-inflammatory drugs, *PSL* prednisolone, *mPSL* methylprednisolone, *CYC* cyclophosphamide, *CQ* chloroquine, *HCQ* hydroxychloroquine, *SASP* salazosulfapyridine, *LEF* leflunomide, *MTX* methotrexate, *ADA* adalimumab, *TAC* tacrolimus, *IVCY* intravenous cyclophosphamide, *ND* not described.

*The RA developed 11 years after the aortitis.

In our patient, the onsets of RA and aortitis occurred at ages 69 and 73 years, respectively, making this patient older than those reported previously. Other reports observed vasculitis findings other than aortitis in at least half of the patients, with findings suggestive of severe RA. Consistent with these observations, our patient had a concurrent skin ulcer. As treatment, PSL was administered at 30 mg/day, a dose comparable to the mean reported steroid dose.

## Discussion

Although we have found case reports on RA-related aortic lesions, this is not yet a widely recognised complication and is considered rare (Towned et al. 1991). By contrast, AS-related aortitis is widely known, and the early literature indicates there was a time when it was difficult to differentiate between aortitis resulting from RA versus AS (Bulkley and Roberts 1973; Clark et al. 1957). It is relatively easy to differentiate aortitis in RA from that in AS based on the clinical course, laboratory results, and radiological tests, even though the two have similar pathological findings (Towned et al. 1991). Lymphoplasmacytic infiltration, necrosis of the medial smooth muscle cells, and elastic fibre loss are findings common to both AS and RA aortitis. In comparison, a rheumatoid granuloma is a characteristic feature that can be clearly differentiated and is seen only in aortitis associated with RA. In addition, TKA and GCA account for many autoimmune aortitis cases. It is reportedly difficult to differentiate the pathological findings of the aorta in these two diseases based on the 2012 CHCC. There are reports of concurrent RA and TKA (Jennette et al. 2013), but like the aortitis from AS, it is difficult to determine whether the aortitis is associated with RA or TKA based on pathology.

RV has been reported to result primarily from vasculitis of small and medium-sized vessels (Genta* et al. 2006). However, many reports have stated that RV occurs in the aorta, a very large vessel (Sandring and Weil 1961; Falicov and Cooney 1964; Reimer et al. 1976; Rush et al. 1986; Sketchler and Waxman 1987; Mimura1 and Sueishi 1989; Gravallese et al. 1989; Towned et al. 1991; Nakabayashi et al. 1998; Korkmaz et al. 2001; Miller et al. 2006; Verweij1 et al. 2012; Mariani and Alexander 2013). RA-associated aortitis essentially occurs in patients with severe RA, indicating an association with RV (Towned et al. 1991). The following risk factors for RV onset have been reported: high-titre rheumatoid factors, course of 1 year or more after the onset, presence of bone erosions, and presence of rheumatoid nodules (Voskuyl et al. 1996). The first two features were observed in our patient. Furthermore, our patient exhibited a skin ulcer, which has been reported in approximately 90% of RV patients (Genta* et al. 2006). However, the patient’s skin biopsy showed no findings suggestive of vasculitis. This might be because the biopsy specimen was obtained from a relatively shallow site; vasculitis might have been detected if the deeper dermal layer had been sampled. It is also entirely possible that the skin ulcer was caused by vasculitis based on the following observations: “no findings suggesting history of trauma, pressure ulcer, or infection”; “vasculitis is the first cause suspected when a deep skin ulcer is observed”, as in this patient; “FDG-PET/CT shows FDG uptakes at the ulcer site, suggesting inflammation”; and “the patient responded well to steroid treatment”. Confirmation that our patient’s skin ulcer resulted from vasculitis would satisfy the Scott and Bacon criteria (Turesson and Jacobsson 2004) for RV, and it is entirely possible that the aortitis in our patient was associated with RV.

A genetic analysis recognised HLA-DRB1*0401 as a risk factor for RV, and approximately 5% and 21% of RV patients are HLA-DRB1*0401 homozygotes and heterozygotes, respectively (Perdriger et al. 1997). HLA-B51 and B52, but not HLA-DRB1*0401, were detected in our case. HLA-B51 (Mizushima 1988) and HLA-B52 (Kimura et al. 2000) are associated with Behçet’s disease and TKA, respectively. These diseases are associated with aortitis. Behçet’s disease was not suspected in our patient, as she did not present the associated findings of oral aphthous ulcers, eye lesions such as uveitis, and genital ulcers. It is unlikely that the aortitis in our case was TKA, as aortitis onset occurred at 74 years of age, while TKA predominantly affects individuals <50 years of age. Furthermore, our patient both met the 2010 ACR/EULAR criteria and experienced interstitial pneumonia, which are suggestive of RA.

Oral PSL at a 30-mg/day dose dramatically improved the clinical findings of aortitis in our patient. Our literature review suggests that the mean PSL dose for aortitis is 46.3 (range 10–100) mg; therefore, a moderate dose of PSL could cure aortitis. Nevertheless, Ellen et al. reported that ten RA-associated aortitis patients died of congestive heart failure, aortic rupture, and acute myocardial infarction; furthermore, most of these patients were not diagnosed with aortitis until after death; therefore, they did not receive any steroid or immunosuppressant therapy. These cases suggest that RA-associated aortitis is a severe disease that can be fatal if left untreated.

## Conclusion

We reported a patient who presented with RV findings, such as cutaneous ulceration and a high-titre rheumatoid factor; the aortitis was associated with RV, and a moderate dose of PSL dramatically improved her clinical findings. The early detection and treatment of RA aortitis is important because RA aortitis can be fatal if left untreated.
